# Comparison of the diagnostic performance of the Swischuk line method and the anterior atlantodental interval method in atlantodental subluxation

**DOI:** 10.1186/s12880-023-01187-z

**Published:** 2024-01-02

**Authors:** Eun Ji Lee, Yeo Ju Kim, Sung Oh Song, Seunghun Lee, Jeongah Ryu, Nayeon Choi

**Affiliations:** 1 Department of Radiology, College of Medicine, Inha University, 27 Inhang-ro, Jung-gu, 22332 Incheon, South Korea; 2 Department of Radiology, Seoul St. Mary’s Hospital, College of Medicine, The Catholic University of Korea, Seoul, South Korea; 3 Department of Radiology, College of Medicine, Hanyang University Hospital, 222, Wangsimni-ro, Seongdong-gu, 04763 Seoul, South Korea; 4 Department of Radiology, Naeun Hospital 23, Wonjeok-ro23, Seo-gu , 22819 Incheon, South Korea; 5 Department of Radiology, College of Medicine, Hanyang University Guri Hospital, 11923 Guri, South Korea; 6 Biostatistical Consulting and Research Lab, Medical Research Collaborating Center, Hanyang University, 222, Wangsimni-ro, Seongdong-gu, 04763 Seoul, South Korea

**Keywords:** Anterior atlantodental subluxation, Anterior atlantodental instability, Anterior atlantodental interval, Swischuk line

## Abstract

**Background:**

Atlantodental subluxation (ADS) is a serious condition that can result in sudden death. Measuring the anterior atlantodental interval (AADI method) is the gold standard for diagnosis but the complex anatomy of this region can make diagnosis difficult, especially for beginners. Therefore, we would like to use a simpler method, the Swischuk line method, to diagnose ADS. The purpose of our study was to evaluate the diagnostic performance of the Swischuk line method for ADS on lateral cervical spine radiographs compared to the AADI method.

**Methods:**

A retrospective study was conducted with patients who presented with ADS (ADS group, n = 32, mean age 57.78 years, age range 34–82 years, 10 men, 21 women) and an age- and sex-matched control group (n = 32). The diagnostic performance of the AADI method and the Swischuk line method for ADS was assessed using lateral cervical radiographs in both flexion and neutral postures by an experienced musculoskeletal radiologist (reader 1), a senior resident (reader 2), and a junior resident (reader 3) in the radiologic department.

**Results:**

In the flexion posture, the AADI method and the Swischuk line method showed excellent diagnostic performance with AUCs > 0.9 for readers 1 2 and reader 3. In a neutral posture, the diagnostic performance of the AADI and Swischuk line methods was decreased. With a 1 mm cut-off value using the Swischuk line method in flexion posture, the sensitivity was 75% or more, the specificity was 100%, and the accuracy was 87.50% or more 90.63% for all readers. With a 2 mm cut-off value, the sensitivity was low (37.50-46.88%) but the specificity was 100% for all three readers. In a neutral posture, the sensitivity for both methods decreased, though specificity remained high (> 80%).

**Conclusions:**

The Swischuk line method was found to be reliable and showed high sensitivity and specificity with a cut-off value of 1 mm for the diagnosis of ADS in cervical lateral radiographs in flexion posture. It can be used as a complement to the AADI method.

**Supplementary Information:**

The online version contains supplementary material available at 10.1186/s12880-023-01187-z.

## Background

Atlantodental subluxation (ADS) is most commonly developed in patients with rheumatoid arthritis (RA), but it can also result from traumatic, inflammatory, or congenital abnormalities [1]. The diagnosis of ADS is essential, as it may be asymptomatic yet can lead to spinal cord compression, vascular compression, and sudden death [1]. Particularly during anesthesia, avoiding unprotected manipulation of the neck is crucial if patients have ADS [2]. Radiography of the cervical spine is the predominant imaging technique for screening ADS, mainly owing to its availability and relatively low cost [1]. The anterior atlantodental interval (AADI) method is the most frequently used diagnostic method for ADS [1, 3]. However, the AADI method has displayed poor interobserver agreement between experienced and novice radiologists, largely due to the complex anatomy of the atlas and axis [2]. As a result of the difficulty in measurement on imaging and the nonspecific symptoms, ADS poses challenges for both radiologists and clinicians [1].

The Swischuk line is a line drawn on a lateral cervical spine radiograph that assists in differentiating between pathological anterior displacement of the 2nd cervical vertebra (C2) on the 3rd cervical vertebra (C3) and physiological displacement, termed pseudo subluxation [4]. Measuring the Swischuk line involves drawing a line from the anterior aspect of the posterior arch of C1 to the anterior aspect of the posterior arch of C3. The anterior aspect of the spinolaminar junction of C2 should be within 1–2 mm of this line (Swischuk line method) [4]. The Swischuk line is a straightforward and easy-to-use method for evaluating the cervical spine in children [4]. Since the Swischuk line method assesses the alignment of the atlas (C1), dens (C2), and C3, we hypothesized that it could also reveal abnormalities in adult ADS. To the best of our knowledge, no studies have tested the diagnostic performance of the Swischuk line in diagnosing ADS in adults. Therefore, our study aims to evaluate the diagnostic performance of the Swischuk line method for atlantodental subluxation (ADS) on lateral cervical spine radiographs compared to the anterior atlantodental interval (AADI) method.

## Methods

### Patient enrollment

The institutional review board of Inha University Hospital approved this study (IRB No: 2020-01-021). Given the retrospective nature of this investigation and the use of anonymized patient data, requirements for informed consent were waived.

#### Atlantodental subluxation (ADS) group

**Fig. 1:**
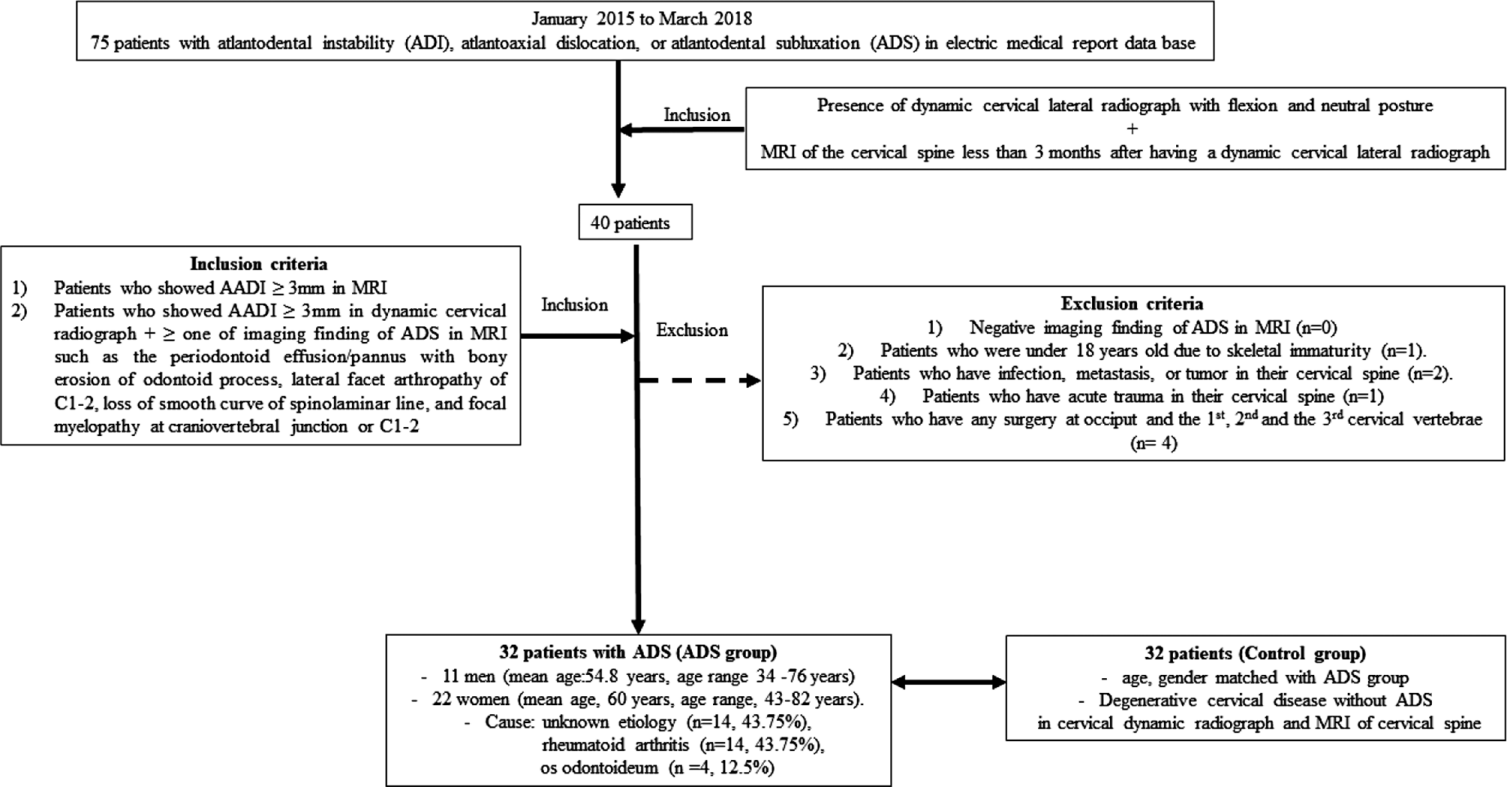
Flowchart of patient inclusion and exclusion criteria

Our electronic medical report database was searched for the period from January 2015 to March 2018 for patients who were reported with atlantoaxial instability, atlantoaxial dislocation, or atlantoaxial subluxation, yielding 75 consecutive patients. Of these patients, 40 performed dynamic cervical lateral radiographs with flexion and neutral posture and had an MRI less than 3 months after having a dynamic cervical lateral radiograph. A musculoskeletal radiologist reviewed the dynamic plain radiographs and MRI of these patients, selecting them according to the following final inclusion and exclusion criteria (Fig. 1):

Inclusion criteria.

1. Patients who showed an atlantodental interval (AADI) of 3 mm or more in MRI.
2. Patients who displayed AADI of 3 mm or more in any position of cervical radiograph with more than one imaging finding of ADS in MRI, such as periodontoid effusion/pannus with bony erosion of the odontoid process, lateral facet arthropathy of C1-2, loss of smooth curve of the spinolaminar line, or focal myelopathy at craniovertebral or C1-2 junction [1].

Exclusion criteria.

1. Patients who did not show any imaging findings of ADS in MRI [1].
2. Patients under 18 years old due to skeletal immaturity.
3. Patients who have an infection, metastasis, or tumor in their cervical spine.
4. Patients who have acute trauma in their cervical spine.
5. Patients who have undergone surgery at the occiput or the 1st, 2nd, or 3rd cervical vertebrae.

According to these inclusion and exclusion criteria, 32 patients were enrolled in our ADS group, comprising 11 men (mean age: 54.8 years; age range: 34–76 years) and 22 women (mean age: 60 years; age range: 43–82 years). The causes of ADS were unknown etiology (n = 14, 43.75%), rheumatoid arthritis (n = 14, 43.75%), and Os odontoideum (n = 4, 12.5%) (Fig. 1).

#### Control group

Our electronic medical report database was searched for the period from January 2018 to March 2018 for the control group who were reported with degenerative cervical disease. Among them, the inclusion criteria for the control group were patients who showed negative AADI method in cervical radiographs with flexion and neutral posture and MRI without any bony or soft tissue abnormality in the atlantoaxial joint. Patients with prior surgery, trauma, tumor, infection, or congenital anomaly, and those under 18 years old, were also excluded. Finally, a control group (n = 32) was selected one by one matching the same age and gender of the ADS group using a software program (MedCalc Software; MedCalc).

## MRI protocol

All MRI of the cervical spine were obtained using a 3.0T MR machine (Discovery MR750w, GE Medical Systems, Milwaukee, Wisconsin, USA). For an sagittal plane, following sequences are conducted: T1- fluid attenuated inversion-recovery (FLAIR) with a repetition time (TR) of 1362 milisecond (ms) and echo time (TE) of 24 ms; T2 weighted fast spin echo (T2-FSE) with a TR of 2800 ms and TE of 90 ms; and T2 weighted fast spin echo (T2-FSE) with iterative decomposition of water and fat with echo asymmetry and least-squares estimation fat saturation (IDEAL) which have a TR of 2250ms and TE of 84.7ms. All sagittal images have a flap angle of 142°, section thickness/gap of 3/0.3 mm, and a field of view of 260 × 260 mm. For an axial plane, T2 FSE with a TR of 5568 ms, TE of 86.8 ms, and a flap angle of 142°; and multiple echo recombined gradient echo (MERGE) sequence with a TR of 550 ms, TE of 13.4 ms and flip angle of 20° were done for disc levels with section thickness/gap of 3/0.3 mm, and field of view of 160 × 160 mm. The total scan time was 17 min and 17 s.

## Cervical radiography protocol

The patient stands in a lateral position with their shoulder against a vertical cassette holder. The detector is positioned in portrait orientation, running parallel to the long axis of the cervical spine on the patient’s left side. The shoulders should be adjusted to lie in the same horizontal plane, ensuring that the patient’s body is in a true lateral position with the cervical vertebrae’s long axis parallel to the plane of the cassette. For a neutral posture, patients are instructed to slightly elevate their chin and lower their shoulder. For a flexion posture, the patient is asked to depress their chin as much as they can tolerate. The central ray (CR) should be perpendicular to the cassette and directed horizontally to the C4 level (the upper margin of the thyroid cartilage). The detector size is 24 × 30 cm, and the x-ray exposure settings range from 50 to 75 kVp and 20 to 40 mAs, with the use of a grid.

## Image analysis

**Fig. 2:**
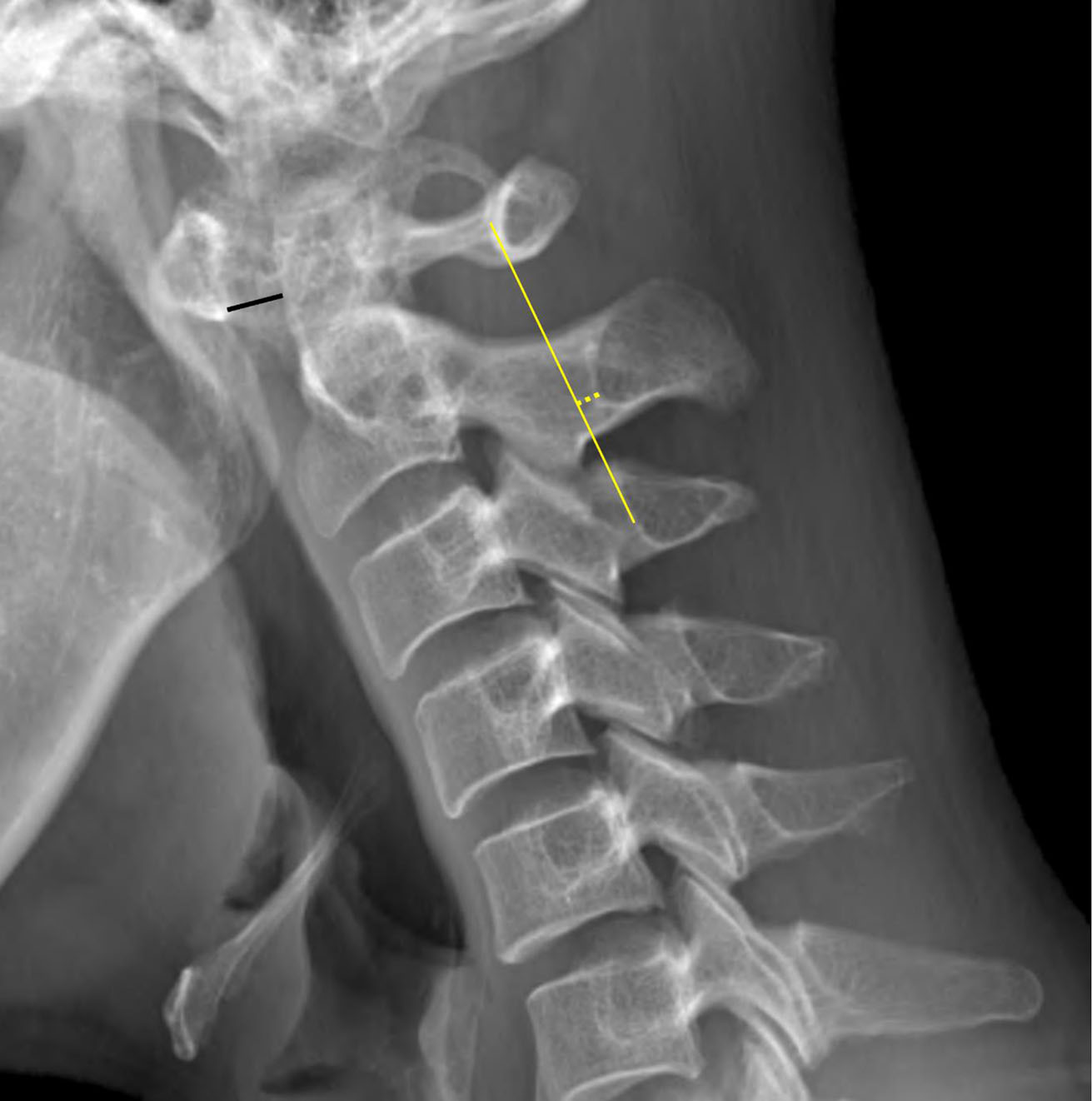
Measurement of AADI Method and the Swischuk Line Method. The AADI method is defined as the measurement of the distance between the posteroinferior aspect of the anterior arch of C1 and the most anterior aspect of the odontoid process (depicted by a black line). The Swischuk line method is defined as the measurement of the distance (depicted by a yellow dashed arrow) from a line drawn from the anterior aspect of the posterior arch of C1 to the anterior aspect of the spinolaminar junction of C3 (Swischuk line, depicted by a yellow line) to the spinolaminar junction of C2 (yellow dashed line). If the spinolaminar junction of C2 is located dorsal to the Swischuk line, as in this case, it is documented as positive values, whereas if the spinolaminar junction of C2 is located ventral to the Swischuk line, it is documented as a negative value

Imaging analysis was conducted for the lateral plain radiograph of the cervical spine with neutral and flexion posture. The lateral cervical radiographs in ADS and control groups were mixed and randomized, then presented separately on workstations after de-identification for blind review. One board-certified musculoskeletal radiologist with 10 years of experience (reader 1), a fourth-grade senior resident (reader 2), and a first-grade junior resident (reader 3) in the radiology department independently measured the ADI and the distance of the spinolaminar junction of C2 to the Swischuk line (Swischuk line method) in all cervical radiographs with neutral and flexion posture. The AADI method was defined as a measurement of the distance between the posteroinferior aspect of the anterior arch of C1 and the most anterior aspect of the odontoid process (Fig. 2). The Swischuk line method was defined as a measurement of the distance from a line drawn from the anterior aspect of the posterior arch of C1 to the spinolaminar junction of C3 (Swischuk line) to the spinolaminar junction of C2 (Fig. 2). If the spinolaminar junction of C2 was located dorsal to the Swischuk line, it was documented as a positive value; whereas if the spinolaminar junction of C2 was located ventral to the Swischuk line, it was documented as a negative value. The definition of ADS in the AADI method was that AADI was to be 3 mm or more [2–4]. In the Swischuk line method, diagnostic cut-off values of both 1 and 2 mm were applied and compared [5]. To evaluate intraobserver agreement. seven months later, three reviewers re-assessed ADS with the AADI method and the Swischuk line method in same manner.

## Statistical analysis

To assess reliability, intraobserver agreement was tested between two repeated measurements per reader. Interobserver agreement was assessed between three readers using the average of the two repeated measurements. Both intra and interobserver agreement were used the interclass coefficient. Based on the 95% confidence interval of the interclass correlation coefficient (ICC) estimate, a κ values less than 0.5 are indicative of poor reliability, values between 0.5 and 0.75 indicate moderate reliability, values between 0.75 and 0.9 indicate good reliability, and values greater than 0.90 indicate excellent reliability [6, 7].

Diagnostic performance was tested using a comparison of the Receiver Operating Characteristic (ROC) curve with post hoc power calculation. The ROC curve and the area under the ROC curve (AUC) were calculated. The accuracy classification by AUC for a diagnostic test is as follows: An AUC of 0.91-1.00 is excellent; 0.81–0.90, very good; 0.71–0.80, good; 0.61–0.70, sufficient; 0.51–0.60, bad; an AUC less than 0.5 was interpreted as not useful [8]. The ROC curves of the AADI method and the Swischuk line method obtained in flexion and neutral postures were analyzed in pairs using the z test [8, 9]. Optimal cutoff values were evaluated by using the maximum Youden index (sensitivity + specificity − 1) for Swischuk line method per each reader [10].

According to the definition of ADS in each method, the AADI and Swischuk line method were statistically analyzed for their diagnostic performance, including sensitivity, specificity, positive predictive value (PPV), negative predictive value (NPV), and accuracy with a 3 mm of cut off value for AADI method and a 1 mm and a 2 mm cut off value for Swischuk line method.

All statistical analyses were performed using commercially available software (SPSS, version 29; IBM, Armonk, NJ, USA) and MedCalc (MedCalc Software; MedCalc) except post hoc power calculation. Post hoc power calculation by R version 4.3.0 (R Foundation for Statistical Computing, Vienna, Austria). For all studies, a difference with a *p*-value less than 0.05 was considered significant.

## Results

### Reliability

The intraobserver and interobserver agreement of AADI and Swischuk line methods in cervical lateral radiographs, both in flexion and neutral postures, can be seen in Table 1. For intraobserver agreement, both AADI method and Swischuk line method showed good or excellent agreement in all three readers in any position. For interobserver agreement, AAID method showed moderate interobserver agreement between reader 1 and reader 3 in flexion posture, and between reader 3 and the other readers in neutral posture. Otherwise, the interobserver agreement was found good or excellent. Especially, Swischuk line method showed excellent interobserver agreement both in flexion and neutral postures except between the reader 1 and reader 3 in neutral posture.

**Table 1:** Interobserver agreement of AADI and Swischuk line method in cervical lateral radiographs in flexion and neutral posture

			AADI method	Swischuk line method
Intraobserver agreement	Flexion posture	R-1 Vs R1-2	0.98 (*P* < 0.001)	0.98 (*P* < 0.001)
		R2-1 Vs R2-1	0.90 (*P* < 0.001)	0.96 (*P* < 0.001)
		R3-1 Vs R3-1	0.89 (*P* < 0.001)	0.98 (*P* < 0.001)
	Neutral posture	R1-1 Vs R1-2	0.97 (*P* < 0.001)	0.88 (*P* < 0.001)
		R2-1 Vs R2-2	0.97 (*P* < 0.001)	0.95 (*P* < 0.001)
		R3-1 Vs R3-2	0.78 (*P* < 0.001)	0.88 (*P* < 0.001)
Interobserver agreement	Flexion posture	R1 Vs R2	0.86 (*P* < 0.001)	0.97 (*P* < 0.001)
		R2 Vs R3	0.83 (*P* < 0.001)	0.98 (*P* < 0.001)
		R1 Vs R3	0.67 (*P* < 0.001)	0.97 (*P* < 0.001)
	Neutral posture	R1 Vs R2	0.97 (*P* < 0.001)	0.96 (*P* = 0.007)
		R2 Vs R3	0.73 (*P* < 0.001)	0.91 (*P* < 0.001)
		R1 Vs R3	0.74 (*P* < 0.001)	0.86 (*P* < 0.001)

### Diagnostic performance

**Fig. 3:**
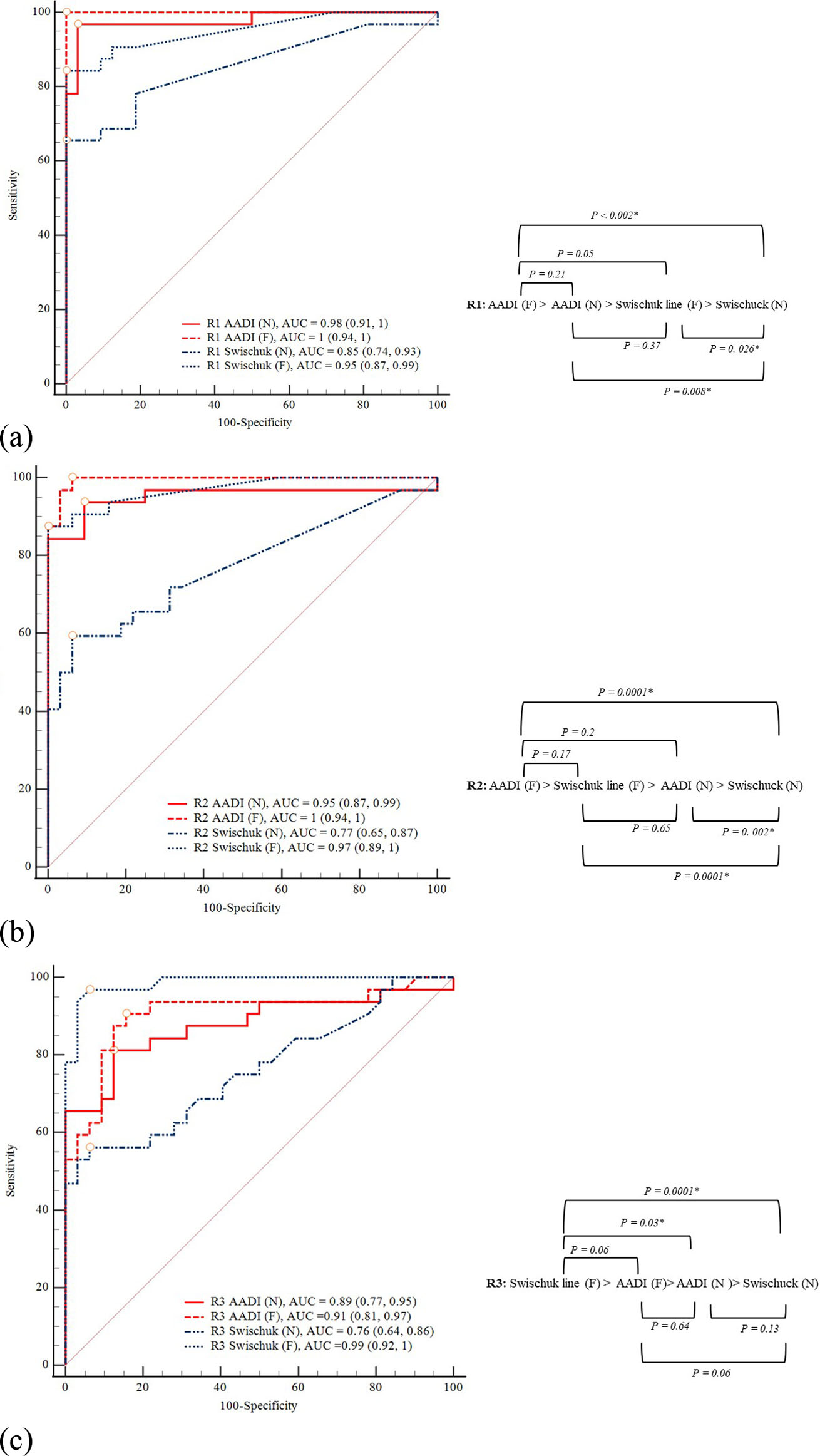
ROC (receiver operating characteristic) curve and the results of pairwise comparison of ROC curves of reader 1 (**a**), reader 2 (**b**) and reader 3 (**c**). Labels: AADI(N) = AADI method in neutral posture; AADI (F) = AADI method in flexion posture; Swischuk (N) = Swischuk line in neutral posture; Swischuk (F) = Swischuk line in flexion posture; AUC = area under curve. Numbers in parentheses indicate the 95% confidence interval.An asterisk (*) indicates statistically significant results (*P* < 0.005). Optimal cutoff values by using the maximum Youden index (sensitivity + specificity − 1) for each ROC curve were represented by an empty circle point

The ROC curves and diagnostic performance of both the AADI and Swischuk line methods, in flexion and neutral postures across three readers, are shown in Fig. 3; Table 2. Post hoc power calculation reveals that the power of each ROC curve for three reviewers ranged from 0.975 to 1.000 which mean sufficient statistical power (Table 2). Both methods displayed a higher AUC in flexion compared to neutral posture (Fig. 3). In the flexion posture, both the AADI method and the Swischuk line method in all three readers showed excellent diagnostic performance (AUCs > 0.9). In reader 1 and 2, the AADI method presented higher AUCs than the Swischuk line method, while the Swischuk line method had a higher AUC than the AADI method in reader 3, but all did not show statistically significant differences. In the neutral posture, the diagnostic performance of the AADI method was excellent in reader 1 and 2 and very good in readers 3. The diagnostic performance of the Swischuk line in the neutral position was very good for reader 1 and good for reader 2 and 3. In neutral posture, the AADI method had higher AUCs than the Swischuk line method in reader 1, with statistical significance but showed no significant differences with the other readers. The optimal Youden index of Swischuk line method for reader 1, 2, and 3 was more than 0.83, 0.64, 0.23 in flexion posture and 0.55, 0.87, 0.84 in neutral posture, respectively.

**Table 2:** The diagnostic performance of the AADI method (AADI) and the Swischuk line method (SL) in flexion and neutral posture of three readers

	Posture	Method	Post-hoc power	Cut-off value	Control group	AAS group	*P value*	Sensitivity (%)	Specificity (%)	PPV (%)		NPV (%)		Accuracy (%)
R1	Flexion	AADI	Not available due to AUC = 1	< 3 mm > 3 mm	32 0	2 30	< 0.001	93.75	100	100		94.12		96.88
		SL	1	< 1 mm > 1 mm	32 0	7 25	< 0.001	78.13	100	100		82.05		89.06
				< 2 mm > 2 mm	*32* *0*	17 15	< 0.001	46.88	100	100		65.31		73.44
	Neutral	AADI	1	< 3 mm > 3 mm	*32* *0*	11 21	*< 0.001*	65.63	100	100		74.42		82.81
		SL	0.999	< 1 mm > 1 mm	*32* *0*	12 20	*< 0.001*	62.50	100	100		72.73		81.25
				< 2 mm > 2 mm	*32* *0*	23 9	*< 0.001*	28.13	100	100		58.18		64.02
R2	Flexion	AADI	1	< 3 mm > 3 mm	31 1	1 31	< 0.001	96.88	96.88	96.88		96.88		96.88
		SL	1	< 1 mm > 1 mm	32 7	7 25	< 0.001	75.00	100	100		80		87.5
				< 2 mm > 2 mm	*32*	20	< 0.001	37.50	100	100		61.54		68.75
					*0*	12								
	Neutral	AADI	1	< 3 mm > 3 mm	*32* *0*	12 20	*< 0.001*	62.50	100	100		72.73		81.25
		SL	0.982	< 1 mm > 1 mm	*30* *2*	14 18	*< 0.001*	56.25	93.75	90		68.18		75
				< 2 mm > 2 mm	*32* *0*	24 8	*< 0.001*	25.00	100	100		57.14		62.50
R3	Flexion	AADI	1	< 3 mm > 3 mm	29 12	3 20	< 0.001	62.5	90.63	86.96		7.73		76.56
		SL	1	< 1 mm > 1 mm	32 0	8 24	< 0.001	75.00	100	100		80.00		87.50
				< 2 mm > 2 mm	*32* *0*	19 13	< 0.001	40.63	100	100		62.75		70.31
	Neutral	AADI	0.999	< 3 mm > 3 mm	*32* *0*	21 11	*< 0.001*	34.38	100	100		60.38		67.19
		SL	0.975	< 1 mm > 1 mm	*30* *2*	14 18	*< 0.001*	56.25	93.75	90		68.18		75
				< 2 mm > 2 mm	*32* *0*	25 7	*0.005*	21.88	100	100	56.14		60.94	

*Note*: PPV = positive predictive value, NPV = negative predictive value, R1 = reader 1, R2 = reader 2, R3 = reader 3, AADI = AADI method, SL = Swischuk line method

**Fig. 4:**
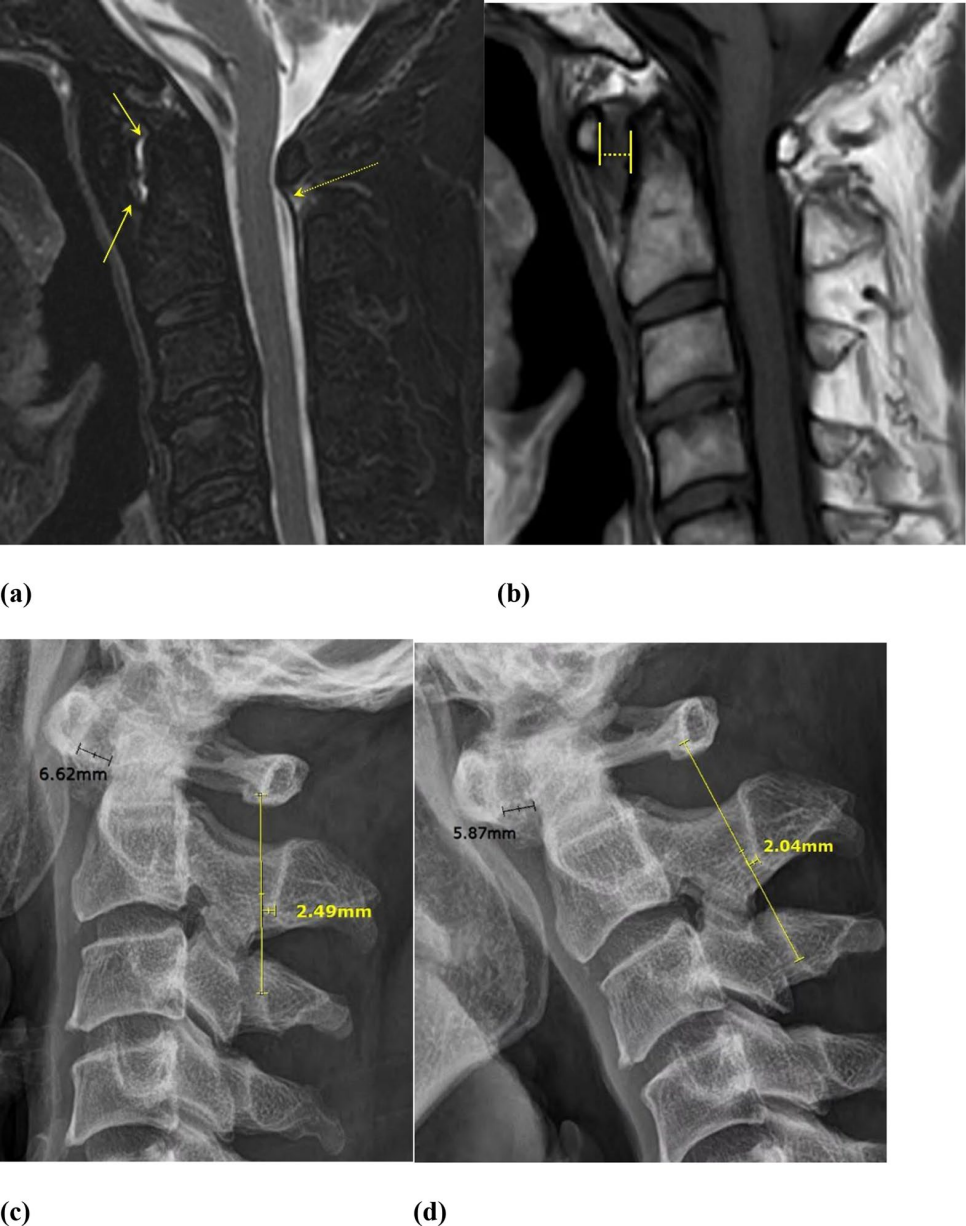
MRI and cervical lateral radiography of a patient with rheumatoid arthritis in ADS group. (**a**) A sagittal T2-weighted fast spin-echo image with iterative decomposition of water and fat using echo asymmetry and least-squares estimation (IDEAL) fat saturation demonstrates high signal intensity at the anterior atlantodental interval (arrows). Additionally, there is disruption of the smooth curvature of the spinolaminar line and edema within the interspinous ligament (dashed arrow). (**b**) A sagittal T1-weighted fast spin-echo image reveals a widened anterior atlantodental interval (4 mm). (**c**, **d**) In lateral cervical radiography with neutral (**c**) and flexion posture (**d**), the anterior atlantodental interval is positive with 6.62 mm in neutral posture and 5.87 mm in flexion posture (cut off value: >3 mm). Swischuk line method is also positive with 2.49 mm in neutral posture and 2.04 mm in flexion posture, applying either 1 or 2 mm cut- off values

**Fig. 5:**
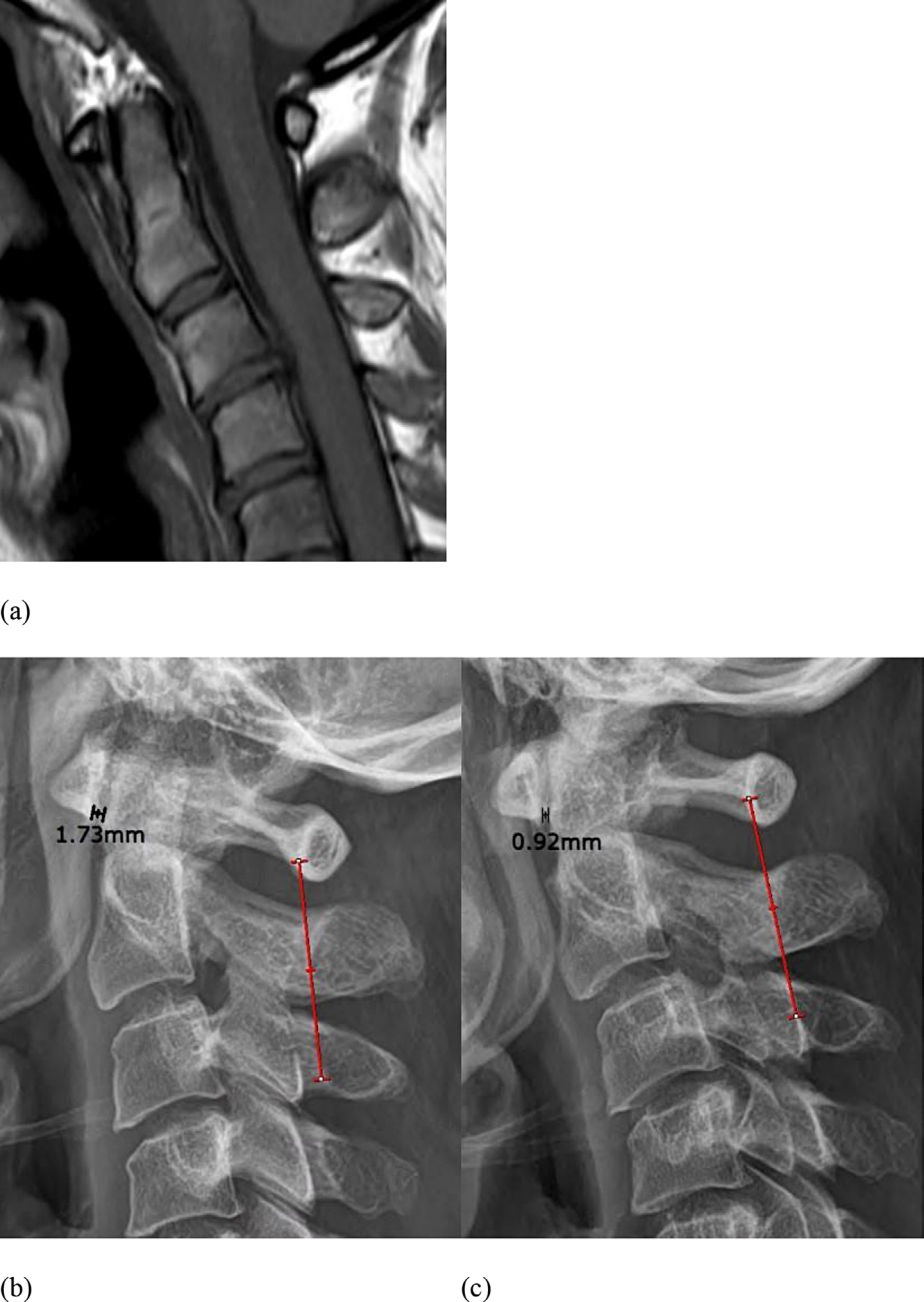
MRI and cervical lateral radiography of a patient in control group. (**a**) Sagittal T1 weighted fat spin echo image shows no abnormality in the atlantoaxial joint with normal range of anterior atlantodental interval. (**b**) In lateral cervical radiography with neutral (**b**) and flexion posture (**c**), the anterior atlantodental interval is measured less than 3 mm, which is negative for AADI method. (1.73 mm in neutral posture, 0.92 mm in flexion posture). The Swischuk line touches the spinolaminar junction of C2 (0 mm in Swischuk line method) in both neutral and flexion posture, which is also negative for Swischuk line method

The sensitivity, specificity, positive predictive value, negative predictive value, and accuracy of the AADI method with a cut-off value of 3 mm and the Swischuk line method with cut-off values of 1 and 2 mm in flexion and neutral postures are illustrated in Table 2. In flexion posture, both methods exhibited high sensitivity, specificity, and accuracy, except for the AADI method in reader 3. Regarding the Swischuk line method, when the cut-off value was set to 1 mm, the sensitivity was 75% or more, the specificity was 100%, and the accuracy was more than 87% for all readers. With a 2 mm cut-off value, the sensitivity was low (37.5-46.88%) for all three readers. In contrast, in the neutral position, both the AADI method and the Swischuk line method demonstrated decreased sensitivity but high specificity (> 93.75%). The range of sensitivity for the AADI method was from 34.38 to 65.63%. The sensitivity of the Swischuk line method in neutral posture with a 1 mm cut-off value ranged from 56.25 to 62.5%, and with a 2 mm cut-off value, it ranged from 21.88 to 28.13%. Example images of the patient group and control group are shown in Figs. 4 and 5.

## Discussion

In our study, both the AADI and Swischuk line methods demonstrated strong diagnostic performance across all three readers in the lateral radiograph of the cervical spine with flexion posture. Notably, the Swischuk line method in flexion posture showed that even a beginner radiologist, unfamiliar with the anatomy of the atlantoaxial joint and cervical radiographs, demonstrated excellent interobserver agreement with experienced radiologists.

For the diagnosis of ADS, radiography of the cervical spine is the standard imaging technique for screening, mainly due to its availability and relatively low cost [1, 6]. However, the diagnostic sensitivity is known to be low, especially in neutral posture, a finding that corresponds with our study [7]. Dynamic radiography, including flexion posture, shows higher diagnostic performance compared to neutral posture, but the possibility of measurement variation still exists [8, 9]. According to prior studies, the measurement of the ADI seemed most reproducible between experienced radiologists, but decreased correlation was observed with resident observers, corresponding with our findings [9]. The accuracy of AADI measurement likely reflects the learning curve involved in mastering measurement techniques and understanding the complex anatomy of the atlantoaxial joint [9]. In particular, many cases causing ADS are accompanied by morphological changes, such as erosion of the C2 odontoid process, spurs, and abnormalities in the overlying facet joints [5, 6, 10]. These bony changes are clearly visible on CT, but on the cervical lateral radiography, the structures overlap each other, obliterating the cortical margin of anterior arch of C1 and the odontoid process [11]. Moreover, patients with osteopenia often show poorly defined cortical margins of the odontoid process, making the cortical margin of the anterior arch of C1 and the anterior margin of the odontoid process often difficult to define on lateral x-rays [8]. Due to these limitations of the AADI method, some studies have reported the necessity for MRI. Although MRI is considered the current gold standard for demonstrating cord compromise due to ADS [5], it is time-consuming and expensive, making it unsuitable as a screening method. CT with multiplanar reconstruction is also beneficial for diagnosing ADS. It offers detailed three-dimensional (3D) information about the bony structure of the atlantoaxial joint. This is especially useful for preoperative planning in cases where MRI is contraindicated [11]. However, the supine positioning during scanning of MRI and CT may further decrease the detection rate or downgrade the severity of subluxation due to gravity [1, 12].

The Swischuk line was first introduced for differentiating between physiological displacement of C2 on C3 (pseudosubluxation) in the pediatric population [4]. Our study showed that the Swischuk line method could be used for the diagnosis of ADS in adult patients. In flexion posture with a cut-off value of 1 mm, it showed high sensitivity and specificity for both experienced radiologists and beginners, with high interobserver agreement. Since the optimal Youden index measured by three different experienced readers was 0.23–0.84, we believe that a cut-off value of 1 mm is reasonable considering the submillimeter measurement error. Our study is also consistent with previous MRI studies that have shown abnormal spinolaminar lines in the upper cervical spine were observed at a significantly higher frequency in patients with AADI widening of 3 mm or more [5]. When a cut-off value of 2 mm was used, the sensitivity was very low, but the specificity and PPV were 100% in any posture. Since ADS can cause severe clinical consequences such as paralysis if a diagnosis is missed, sensitivity is more important than specificity, making it advisable to set 1 mm as the cut-off value. Many conditions causing ADS combine morphological changes of the cortex of the anterior arch of C1 or odontoid process of C2, but the spinolaminar junction is relatively easy to define as there is usually no morphological change except congenital posterior arch anomalies and no overlying anatomical structures. However, in our study, we also observed several limitations with the Swischuk line method. First, among experienced radiologists, the AADI method still showed higher diagnostic performance than the Swischuk line method in lateral radiography in experienced radiologist, especially in neutral posture. It implies that the AADI method remains the best diagnostic method for ADS screening after establishing the learning curve. Second, the Swischuk line method showed decreased diagnostic performance and low reliability with neutral posture. In flexion posture, the spinolaminar junctions of C1, C2, and C3 are usually aligned in a single line and easy to draw. However, in neutral posture, due to the lordotic curve of C1, C2, and C3, the spinolaminar junction may not be aligned in a single line, resulting in variation and measurement error. Kunakornsawat et al. reported great variation in the relative position of the C1 spinolaminar line in the normal population [11]. According to their study, 4.4% of the normal population showed the C1 lamina lying 2 mm ventral to the C3–C2 spinolaminar line in lateral cervical radiographs with neutral posture [11]. They reported that this variation was more prevalent in C1 stenosis cases and had slightly wider AADI compared to the other population, although the AADI was within the normal range, and all patients were asymptomatic [11]. They assumed that this variation might be a degenerative change rather than congenital, as most of the cases were more than 60 years old [11]. Oshima et al. also examined the value of the relative position of the C1 lamina compared to the spinolaminar line connecting between C2 and C3 in patients without atlantoaxial instability [12]. They found a few patients (1.6%) had a smaller space available for the spinal cord at the C1 level than that of the C2 level, and all these patients showed the C1 lamina lying ventral to the C3–C2 spinolaminar line [12]. These previous studies imply that the Swischuk line method might be a false positive as normal variation and without ADS [11, 12]. Therefore, the Swischuk line method may not replace the AADI method and should be used as a complement to the AADI method when diagnostic confidence is low.

Our study has several limitations. First, we included a small study population, meaning a large population, patient-controlled study will be needed in the future. Second, we included only patients confirmed to have ADS by MRI to exclude false-positive cases. Since MRI scans were performed on patients suspected of spinal cord compression or requiring surgical treatment, our study may be subject to selection bias by including only patients with relatively severe ADS. Finally, the causes of ADS were not consistent and varied, which may have affected the diagnostic performance.

## Conclusions

The Swischuk line method was found to be reliable and showed high sensitivity and specificity with a cut-off value of 1 mm for the diagnosis of ADS in cervical lateral radiographs in flexion posture. It can be used as a complement to the AADI method.

### Electronic supplementary material

Below is the link to the electronic supplementary material.

Supplementary Material 1

## Data Availability

The datasets used and/or analyzed during the current study are available from the corresponding author upon reasonable request.

## Abbreviations

ADS: atlantodental subluxation
AADI: anterior atlantodental interval
C1: the first cervical vertebra (atlas)
C2: the second cervical vertebra (dens)
C3: the third cervical vertebra
ICC: interclass correlation coefficient
ROC: Receiver Operating Characteristic
AUC: the area under the ROC curve
